# EFFECTS OF MEDICARE COMORBIDITIES, SELF-REPORTED FACTORS, AND POLYGENIC RISK SCORES IN RISKS OF AD/ADRD

**DOI:** 10.1093/geroni/igz038.1798

**Published:** 2019-11-08

**Authors:** Igor Akushevich, Arseniy Yashkin, Julia Kravchenko, Svetlana Ukraintseva, Anatoliy Yashin

**Affiliations:** 1 Duke University, Durham, North Carolina, United States; 2 Duke University School of Medicine, Durham, North Carolina, United States

## Abstract

At this time there is no consensus on the origin, development, and progression of Alzheimer’s Disease and related dementias (AD/ADRD) and the extent to which variation in the effects of potential risk factors affects the risk for this disorder is underexplored. In this paper we used HRS-Medicare-genetics data to evaluate the effects of risk factors from three groups: i) Medicare-based indicators of chronic diseases that have shown an association with AD/ADRD in the literature, ii) individual heath state, behavior, functional status, education and socioeconomic status, and iii) polygenic risk scores that incorporate known-to-date genetic risk factors for AD/ADRD. We found that: i) the effects of Medicare disease indicators are higher than the effects of self-reported diseases; ii) heart diseases, cerebrovascular diseases, and depression had a strong impact on AD/ADRD, while the presence of cancers sometimes decreases the risk of AD/ADRD; iii) systemic hypotension, chronic kidney disease, and chronic liver disease showed unexpectedly strong effects; iv) compared to females, males are affected by a lower number of risk factors albeit at higher magnitudes; v) BMI, alcohol, drinking, income, and number of education years are protective, vi) genetic scores associated with neurotransmitters (synapse functioning and loss) and neuroinflammation demonstrated strong significant effects, and vii) Blinder-Oaxaca decomposition demonstrated the important role of genetic factors in racial disparities in AD risk. The analyses show the extent to which links between the distinct differences in comorbidities, behavioral and socioeconomic factors can predict the risk for AD/ADRD.

